# Exploring the toxicity mechanisms and detoxification methods of Rhizoma Paridis

**DOI:** 10.1515/biol-2022-1055

**Published:** 2025-11-27

**Authors:** Sun Jianwei, Zhang Guowen, Zhang Yan, Zheng Mengyang, Du Zefei, Liang Haifeng

**Affiliations:** Fifth Affiliated Hospital of Kunming Medical University, Honghe, 661000, P.R. China

**Keywords:** Rhizoma Paridis (Chonglou), toxicity, pharmacological effects, detoxification methods, toxicity assessment

## Abstract

Although there has been research on the toxicity of Rhizoma Paridis (Chong Lou), a systematic and comprehensive evaluation, as well as targeted strategies for mitigating its toxicity, remains lacking. This review aims to provide a thorough assessment of the toxicological properties of Rhizoma Paridis, offering reliable guidance for its safe application. The article focuses on the toxicity and underlying mechanisms of Rhizoma Paridis and reviews the applications of both traditional Chinese medicine and modern scientific technologies in detoxification. Finally, it proposes future research directions, emphasizing the need for further exploration of chronic toxicity mechanisms and advocating for the integration of traditional and modern detoxification methods to ensure the safe clinical use of Rhizoma Paridis. This review provides the latest theoretical foundation for the safe use and further development of Rhizoma Paridis.

## 1 Introduction

Rhizoma Paridis, a traditional Chinese herbal medicine, is derived from the dried rhizomes of Dian Paris (*Paris polyphylla* Smith var. Mazz.) and seven-leaved Paris (*P. polyphylla* Smith var.), both belonging to the genus *Paris* in the Liliaceae family [1,2]. Its medicinal properties are well-documented in classical texts of Traditional Chinese Medicine (TCM), such as the Shennong Bencao Jing [3]. Rhizoma Paridis is particularly recognized for its effects in clearing heat, detoxifying the body, reducing swelling, alleviating pain, cooling the blood, and stopping bleeding [4]. Historically, it has been used for centuries to treat a variety of conditions, including mumps, bleeding disorders, snake bites, fractures, and abscesses [5].

**Figure 1:**
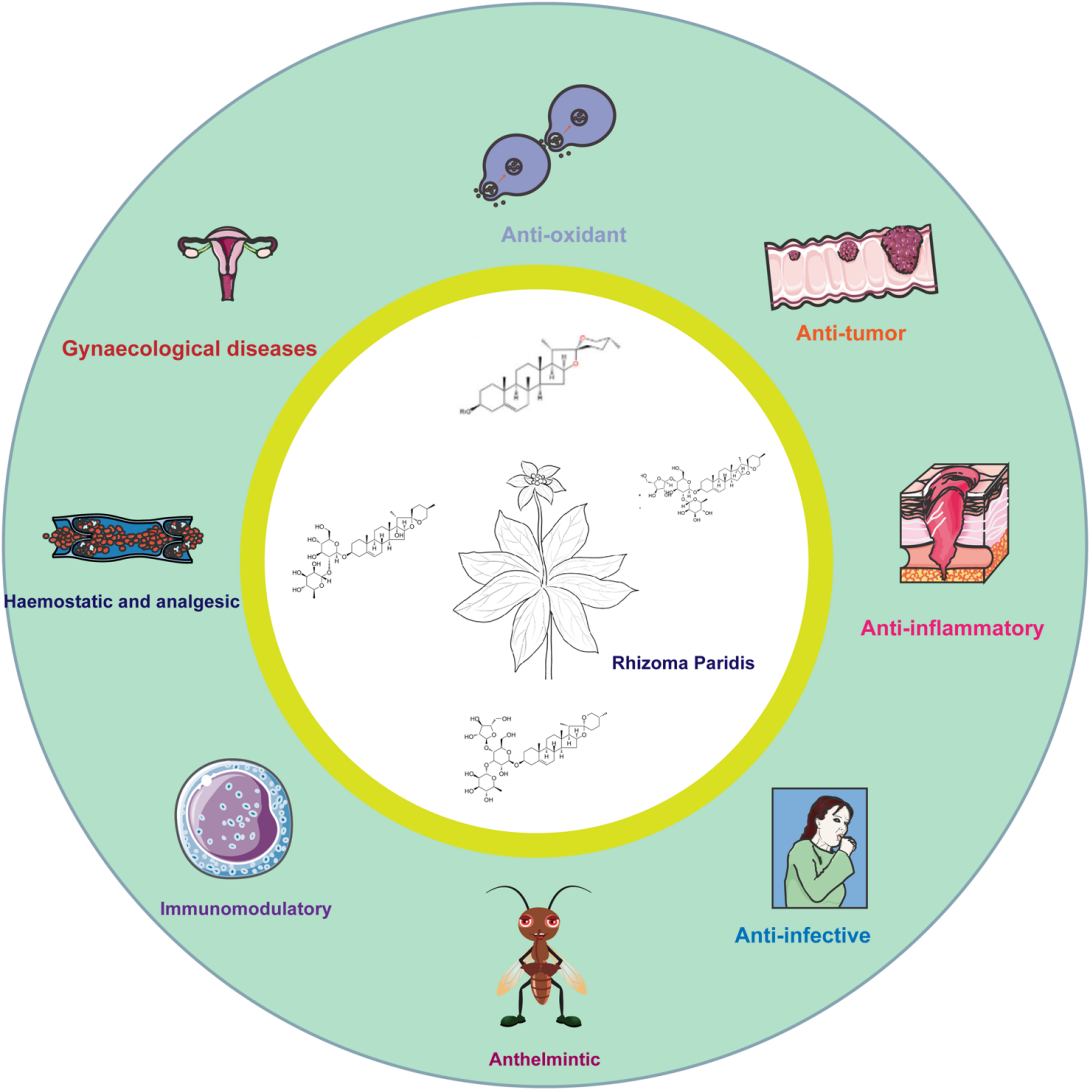
Pharmacological activities of Rhizoma Paridis.

Pharmacological studies have demonstrated that Rhizoma Paridis possesses a range of bioactive properties, including antitumor, antimicrobial, anti-inflammatory, etc. (Figure 1) [6–14]. Due to these pharmacological activities, Rhizoma Paridis is commonly included in TCM formulations for both internal and external use, treating a variety of conditions such as inflammation, tumors, snake bites, and traumatic infections. Additionally, it serves as the raw material for 90 proprietary Chinese medicines and is widely used in clinical practice (Table 1) [15–31].

**Table 1:** Representative proprietary chinese medicines containing Rhizoma Paridis

Category	Drug name	Effects	Clinical indications	**Approval number**
**Analgesics** [15,20]				
Baibao Dan		Dispel blood stasis and reduce swelling, stop bleeding and relieve pain	Knife and gun injuries, traumatic injuries, menstrual irregularities, dysmenorrhea, amenorrhea, chronic gastralgia, and joint pain	Guoyao Zhunzi Z53021108
Changchun hongyao capsule		Promote blood circulation and remove blood stasis, reduce swelling and relieve pain	Traumatic injuries with blood stasis and pain	Guoyao Zhunzi Z20026502
Yili Zhitongwan		Clear heat and detoxify, promote blood circulation, and relieve pain	Pain caused by knife and gun injuries, traumatic injuries, dysmenorrhea in women, and pain associated with advanced malignant tumors	Guoyao Zhunzi Z50020232
Zhongtong Qiwuji		Reduce swelling, alleviate pain, promote blood circulation, remove blood stasis, relax tendons and activate collaterals, resolve masses, and disperse nodules	Traumatic injuries, rheumatic joint pain, frozen shoulder, gouty arthritis, and mammary gland hyperplasia	Guoyao Zhunzi Z20025741
Chanwu Babugao		Promote blood circulation, remove blood stasis, reduce swelling, and relieve pain	Treatment of cancer-related pain; also applicable to acute and chronic sprains, traumatic injuries, bone hyperplasia, rheumatism, and rheumatoid pain, as well as conditions like stiff neck, frozen shoulder, lumbar muscle strain, and injuries	Guoyao Zhunzi Z20027885
Diedabang Yaojiu		Reduce swelling and relieve pain	Traumatic injuries with blood stasis, swelling, and pain	Guoyao Zhunzi Z44023384
Shennong Zhentong Gao		Activate blood circulation, disperse blood stasis, reduce swelling, and relieve pain	Traumatic injuries, rheumatic joint pain, and lumbar and back soreness	Guoyao Zhunzi Z44020387
Xiongdan Diedagao		Activate blood circulation, disperse blood stasis, reduce swelling, and relieve pain	Traumatic injuries, rheumatic joint pain, and lumbar and back soreness	Guoyao Zhunzi Z20026055
**Anticancer drugs** [15,21–24]				
Boerning capsule		Support healthy energy, expel pathogenic factors, replenish qi, activate blood circulation, soften and disperse nodules, reduce swelling, and relieve pain	Adjunctive treatment for cancer	Guoyao ZhunziZ20054459
Ganfule Pian		Strengthen the spleen and regulate qi, resolve blood stasis, soften nodules, and clear heat and detoxify	Primary liver cancer	Guoyao Zhunzi Z10940066
Loulian capsule		Regulate qi, dissolve blood stasis, and clear heat and detoxify	Adjunctive treatment for primary liver cancer	Guoyao Zhunzi Z10980133
Qizhen Capsule		Replenish qi, resolve blood stasis, and clear heat and detoxify	Treatment of cancers such as lung cancer, breast cancer, and gastric cancer	Guoyao Zhunzi Z20010074
Ruan-Jian oral liquid		Resolve blood stasis, soften nodules, detoxify, and replenish qi	Primary liver cancer	Guoyao Zhunzi Z20184027
Xiongdan capsule		Possess the effects of clearing heat and dispersing nodules, activating blood circulation, eliminating masses, and strengthening the body	Adjunctive treatment for nasopharyngeal cancer, esophageal cancer, gastric cancer, and rectal cancer	Guoyao Zhunzi Z19983163
**Gastrointestinal drugs** [15]				
Quyu Yiwei Capsule		Strengthen the spleen and harmonize the stomach, resolve blood stasis, and relieve pain	Gastric pain caused by spleen deficiency, qi stagnation, and blood stasis, as well as symptoms of chronic superficial gastritis	Guoyao Zhunzi Z20174017
Xuedan Weichangwan		Warm the middle, dispel cold, regulate qi, and relieve pain	Cold deficiency in the middle burner causing gastric cold pain, belching, acid regurgitation, loose stools, gastric ulcers, duodenal ulcers, duodenitis, and proctitis	Guoyao Zhunzi Z20025385
**Gynecological drugs** [15,25–27]				
Gongxuening capsule		Cool the blood, stop bleeding, clear heat, eliminate dampness, and relieve pain	Excessive uterine bleeding, heavy menstruation, postpartum or post-abortion uterine atony bleeding, uterine bleeding due to blood heat, and chronic pelvic inflammation causing lower abdominal pain, lumbosacral pain, and increased vaginal discharge	Guoyao Zhunzi Z20020087
Sanjie Zhitonggao		Soften and disperse nodules, reduce swelling, and relieve pain	Cystic hyperplasia of the mammary gland, mastalgia, and gynecomastia in males	Guoyao Zhunzi Z44023492
**Heat-clearing and detoxification** [15,28]				
Hongwei Sheyaopiao		Clear heat, detoxify, reduce swelling, relieve pain, cool the blood, and disperse blood stasis	Used for snake bites and poisonous insect bites	Guoyao Zhunzi Z36021580
Jideshengshe tablet		Clear heat, detoxify, reduce swelling, and relieve pain	Snake and poisonous insect bites	Guoyao Zhunzi Z32020048
Zhanjiang Sheyao		Neutralize snake venom, relieve pain, and reduce swelling	Snake bites, centipede bites, and other poisonous insect stings	Guoyao Zhunzi Z44023712
**Liver-related drugs** [15]				
Biyunsha Yigan Keli		Clear the liver, detoxify, and regulate qi and blood circulation	Hepatitis B caused by liver and gallbladder damp-heat syndrome	Guoyao Zhunzi Z20026322
Caoxian Yigan Capsule		Clear heat, detoxify, strengthen the spleen, and drain dampness	Chronic hepatitis B, flank pain, abdominal distension due to hepatitis B	Guoyao Zhunzi Z20204009
Qinggan Baiduwan		Clear heat, drain dampness, and detoxify	Acute and chronic hepatitis due to liver and gallbladder damp-heat syndrome	Guoyao Zhunzi Z20026098
**Respiratory system drugs** [15,29]				
Xiaoer Tuire Chongji		Dispel wind and release the exterior, detoxify, and relieve throat discomfort	Pediatric cold and fever caused by external wind-heat, as well as upper respiratory tract infections with similar symptoms	Guoyao Zhunzi Z37021424
Fufang Chuanbei Zhiketangjiang		Suppress cough and resolve phlegm, moisten the lungs, and calm asthma	Cold with cough, excessive phlegm, and shortness of breath	Guoyao ZhunziZ44023581
Fufang Shedan Chuanbeisan		Suppress cough and resolve phlegm	Wind-heat cough and chronic cough with excessive phlegm	Guoyao Zhunzi Z44022008
Fufang Yanlian Pian		Clear heat, detoxify, dissolve phlegm, and relieve cough	Cold, cough caused by upper respiratory tract infections, acute and chronic bronchitis, pharyngitis, and tonsillitis	Guoyao Zhunzi Z52020427
Houshu Kouhanpian		Clear heat, detoxify, moisten the lungs, and relieve throat discomfort	Sore throat, throat itching, and dryness	Guoyao Zhunzi Z53020605
Biyan Qingdu Ji		Clear heat, detoxify, reduce inflammation, and disperse nodules	Chronic inflammation of the nasopharynx, sore throat, and increased secretions after radiotherapy for nasopharyngeal cancer	Guoyao Zhunzi Z44022386
Qingre Zhike Keli		Clear heat, resolve phlegm, open the lungs, and relieve cough	Cough with phlegm or heat-related cough	Guoyao Zhunzi Z20000016
Redu Qingpian		Heat-clearing detoxification tablets: clear heat, detoxify, reduce swelling, and disperse nodules	Parotitis, tonsillitis, laryngitis, and upper respiratory tract infections caused by internal heat toxins	Guoyao Zhunzi Z53020800
Shenbei Zhike Keli		Clear the lungs, dissolve phlegm, and relieve cough	Chronic bronchitis with cough	Guoyao Zhunzi Z20026126
Jiuwei Shaungjie Koufuye		Release the exterior and clear heat, purge fire, and detoxify	Wind-heat colds	Guoyao Zhunzi Z20240002
Lanhua Kening Pian		Treat wind-heat attacking the lungs, clear heat, detoxify, astringe the lungs, and relieve cough	Acute and chronic bronchitis, chronic cough, and scanty phlegm	Guoyao Zhunzi Z20043682
Xiaoer Qingreling		Clear heat, detoxify, relieve throat discomfort, and stop coughing	Cold with fever, sore throat, cough, and shortness of breath	Guoyao Zhunzi Z22022819
Rendong Ganmao Keli		Clear heat and detoxify	Fever and throat pain caused by upper respiratory tract infections	Guoyao Zhunzi Z20025083
**Rheumatology drugs** [15]				
Gufengning capsule		Detoxify, dissolve blood stasis, and activate collaterals to relieve pain	Rheumatoid arthritis and ankylosing spondylitis	Guoyao Zhunzi Z20026229
Lujin Zhuanggu Jiu		Dispel wind and dampness, relax muscles, and activate blood circulation	Numbness of limbs and rheumatic arthritis	Guoyao Zhunzi Z22024875
Xuanqi Tongbi Capsule		Nourish and tonify the liver and kidneys, activate blood circulation, remove stasis, reduce swelling, and relieve pain	Rheumatoid arthritis	Guoyao Zhunzi Z19990041
Jingutengtong Jiu		Expel wind and dampness, relax muscles, and activate blood circulation	Muscular and joint pain, limb numbness, and rheumatic arthritis	Guoyao ZhunziZ34020072
**Skin, eczema, and dermatitis drugs** [15,30]				
Jianghuang Xiaocuo Chaji		Clear heat, eliminate dampness, and promote blood circulation to treat acne	Acne (pimples)	Guoyao Zhunzi Z20025149
Yinbing Xiaocuoting		Clear heat, detoxify, cool the blood, and reduce swelling	Acne	Guoyao Zhunzi Z20025294
Keyangminxu		Astringe to stop itching, reduce inflammation, and detoxify	Acute and chronic eczema, urticaria, insect-bite dermatitis, contact dermatitis, and skin itching	Guoyao Zhunzi Z44022982
Chonglou Jieduting		Clear heat, detoxify, and relieve pain by dispersing blood stasis	Herpes zoster, skin itching, insect-bite dermatitis, and epidemic parotitis	Guoyao Zhunzi Z20025808
Xiaozhi Jiefu Ruangao		Clear heat, detoxify, resolve blood stasis, reduce swelling, and eliminate dampness to stop itching	Athlete's foot, body ringworm, groin ringworm, damp sores, internal and external hemorrhoids with swelling, pain, and bleeding caused by damp-heat accumulation	ZF018200
**Trauma and hemostasis** [15,31]				
Yunnan Baiyao		Stop bleeding, relieve pain, reduce inflammation, and promote wound healing	Small open wounds in surgery	Guoyao Zhunzi Z53021102
Shangyi Qiwuji		Reduce swelling, relieve pain, stop bleeding, and disperse blood stasis	Traumatic injuries and mild burns or scalds	Guoyao Zhunzi Z20026238
Yunnanhongyao capsule		Stop bleeding and relieve pain, promote blood circulation, disperse blood stasis, and expel wind and dampness	Gastric ulcer bleeding, bronchiectasis with hemoptysis, functional uterine bleeding, menorrhagia, fundus hemorrhage, conjunctival hemorrhage, epistaxis, hemorrhoidal bleeding, soft tissue contusions, rheumatic arthritis, and rheumatic lumbar-leg pain	Guoyao Zhunzi Z53020129
Sanqi Xueshangning Capsule		Stop bleeding and relieve pain, remove stasis, and promote tissue regeneration	Gastric and duodenal ulcer bleeding, bronchiectasis bleeding, pulmonary tuberculosis with hemoptysis, functional uterine bleeding, trauma and hemorrhoidal bleeding, menstrual disorders, dysmenorrhea, amenorrhea, excessive menstrual bleeding, postpartum blood stasis, gastric disorders, and intercostal neuralgia	Guoyao Zhunzi Z45020612
**Urinary system drugs** [15]				
Jiedutonglin Wan		Clear heat, eliminate dampness, and promote urination	Non-gonococcal urethritis caused by lower burner damp-heat	Guoyao Zhunzi Z20025507
Niaoqingshu Keli		Clear heat, drain dampness, and promote urination	Acute and chronic prostatitis, urinary tract infections, cystitis, and other urological conditions	Guoyao Zhunzi Z20026440
**Others** [15]				
Waiyong Wudigao		Expel wind, remove dampness, activate blood circulation, reduce swelling, relieve pain, clear heat, detoxify, and relieve obstruction and pain	Traumatic injuries, rheumatic numbness, lumbar and shoulder pain, and abscesses with redness, swelling, and pain	Guoyao Zhunzi Z53020661
Weisheng San		Remove filth, clear heat, detoxify, relieve spasms, and calm the mind	High fever with coma, neck stiffness with convulsions, stroke with phlegm retention causing jaw clenching and phlegm obstruction, pediatric convulsions, acute gastroenteritis, vomiting, diarrhea, abscesses, and carbuncles	Guoyao Zhunzi Z21022012

Despite its significant medicinal benefits, the potential toxicity of Rhizoma Paridis must not be overlooked. Research has identified several chemical compounds, including steroidal saponins and alkaloids, which contribute to its therapeutic effects but may also trigger toxic reactions [32]. Therefore, it is crucial to understand the toxic constituents and the mechanisms underlying their toxicity to ensure the safe use of Rhizoma Paridis in medical practice. Comprehensive studies on its toxicity, as well as strategies for prevention and detoxification, are essential not only for advancing the modernization of traditional medicine but also for promoting the safe and rational application of Rhizoma Paridis.

This study provides a systematic examination of the chemical composition, toxicological profiles, and mechanisms of toxicity associated with Rhizoma Paridis. It further investigates prevention and detoxification strategies, methods of toxicity assessment, and potential directions for future research. The findings aim to offer a comprehensive framework for ensuring the safe application of Rhizoma Paridis in clinical practice and guiding further scientific exploration.

## 2 Chemical composition of Rhizoma Paridis

Rhizoma Paridis contains a diverse range of natural compounds. To date, more than 200 chemical constituents have been isolated and identified from Rhizoma Paridis [33–35].

### 2.1 Steroidal saponins

Steroidal saponins are the primary active compounds in Rhizoma Paridis, renowned for their diverse biological activities [36,37]. Their chemical structure, which resembles that of steroid hormones, allows them to exert various pharmacological effects in the body. To date, 136 distinct steroidal saponins have been isolated and identified from Rhizoma Paridis, with spirostanol and furostanol being the most predominant, together accounting for approximately 80% of the total saponins [38–42]. Notable examples of these saponins include Polyphyllin I (PPI), Polyphyllin II (PPII), and Polyphyllin VII (PPVII).

### 2.2 C21 steroids

C21 steroids have attracted considerable attention due to their distinctive chemical structures and a wide range of pharmacological effects, including antitumor, anti-inflammatory, sedative, and analgesic properties [43–45]. As key constituents of Rhizoma Paridis, these compounds are responsible for many of its biological activities [46].

### 2.3 Flavonoid

Flavonoids are lipophilic constituents of Rhizoma Paridis [47]. Although present in relatively low concentrations, these compounds demonstrate notable antioxidant and anti-inflammatory activities. Their effects are primarily mediated through the scavenging of free radicals and the inhibition of inflammatory mediator release [48].

### 2.4 Others

In addition to the aforementioned components, Rhizoma Paridis contains other classes of compounds, including plant growth regulators, plant sterols, quinones, fatty acids, alkaloids, and phenylpropanoids, as identified in relevant pharmaceutical research [49].

## 3 Toxicological studies on Rhizoma Paridis

Although Rhizoma Paridis plays a significant therapeutic role in clinical practice, there remains a limited understanding of its toxicity and the underlying mechanisms of its toxic effects. This section reviews and analyzes previous studies to provide a comprehensive understanding of the toxicological properties of Rhizoma Paridis, offering a theoretical foundation and data support for future research.

### 3.1 Hepatotoxicity

The liver is the primary organ for drug metabolism in the body and is also a key target for drug-induced toxicity [50–52]. Hepatotoxicity is a common cause of liver damage, manifesting in a variety of clinical symptoms [53,54]. A thorough understanding of the hepatotoxic effects of TCMs is crucial, particularly in the context of new drug development and clinical drug use, to ensure safety assessments and minimize drug-induced liver damage. The following sections will discuss in detail the clinical manifestations and specific mechanisms by which Rhizoma Paridis saponins induce hepatocellular injury.

#### 3.1.1 Clinical manifestations

Rhizoma Paridis is a toxic herb, and its hepatotoxicity exhibits a clear dose-dependent relationship. Experimental studies have shown that when the total saponin dose exceeds 4–5 times the recommended amount, significant liver damage occurs. For instance, in a zebrafish model, high doses of Rhizoma Paridis saponins caused scattered necrosis in liver tissue, disrupted hepatocyte arrangement, and triggered apoptosis and vacuolation. Additionally, in rat models, when the total saponin dose exceeded 265 mg/kg (approximately five times the equivalent dose for humans), noticeable hepatocellular damage was observed. Histopathological analysis of liver tissue revealed the disappearance of hepatocyte membranes, cell enlargement, prominent binucleation, and severe nuclear fragmentation. These pathological changes further confirm the hepatotoxic effects of Rhizoma Paridis saponins [55].

#### 3.1.2 Mechanisms of hepatotoxicity

##### 3.1.2.1 Induction of apoptosis

**Figure 2:**
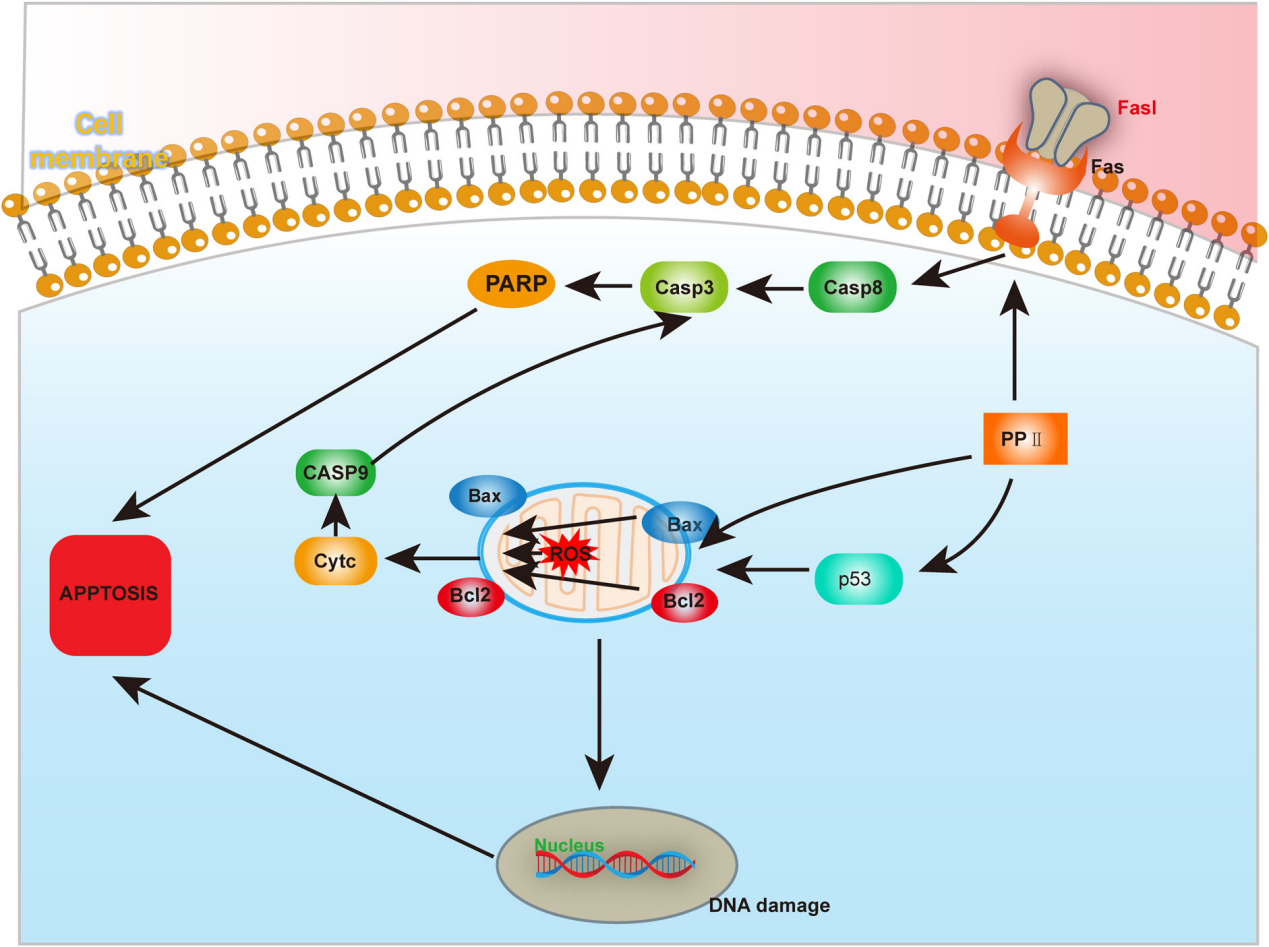
The mechanism of Rhizoma Paridis saponin-induced apoptosis in hepatocytes.

Apoptosis is a key mechanism underlying liver injury induced by Rhizoma Paridis. Studies have shown that Rhizoma Paridis triggers hepatocyte apoptosis by activating both intrinsic (mitochondrial) and extrinsic (death receptor) apoptotic pathways. These pathways ultimately converge on a caspase cascade, leading to hepatocellular death. Additionally, oxidative stress and the generation of reactive oxygen species (ROS) exacerbate the apoptotic process (Figure 2) [56,57].

- Intrinsic pathway: Mitochondrial pathway
  Mitochondria are the primary source of ROS within cells. PPⅡ stimulates the production of ROS in mitochondria, inducing oxidative stress. Excessive ROS not only directly damage cellular lipids, proteins, and DNA, but also activate the tumor suppressor protein p53. p53 serves as a DNA damage sensor, and in response to ROS, it upregulates the pro-apoptotic protein Bax while downregulating the anti-apoptotic protein Bcl-2, altering the Bax/Bcl-2 ratio. This disruption leads to a loss of mitochondrial membrane potential (Δ*ψm*), increased mitochondrial membrane permeability, and the release of cytochrome c into the cytoplasm. Cytochrome c activates downstream caspases, including caspase-9 and caspase-3, which ultimately result in hepatocyte apoptosis. ROS play a crucial role in amplifying liver damage by potentiating this mitochondrial apoptotic pathway [58,59].
- Extrinsic pathway: Death receptor pathway
  In the extrinsic apoptotic pathway, the binding of Fas (death receptor 1) to its ligand FasL initiates the activation of caspase-8. Caspase-8 subsequently activates caspase-3 and cleaves poly (ADP-ribose) polymerase, further promoting apoptosis. PPII have been shown to upregulate Fas, Bax, and cytochrome c expression, while activating caspase-3, caspase-8, and caspase-9 in a dose- and time-dependent manner, thereby enhancing hepatocyte apoptosis [59].

##### 3.1.2.2 Impact on cytochrome P450 (CYP450) enzymes

CYP450 enzymes are essential metabolic enzymes in hepatocytes, playing a key role in the metabolism of most drugs by enhancing their polarity and water solubility [60–62]. Upon entering the body, PPI can influence the CYP450 enzyme system, leading to alterations in the expression of drug-metabolizing enzymes such as CYP1A1, CYP1A2, and phase II enzymes like GSTA3. These changes in enzyme expression may disrupt normal drug metabolism, potentially contributing to liver damage.

##### 3.1.2.3 Hepatic energy and lipid metabolism imbalance

Dysfunction in mitochondrial respiratory chain reactions and lipid metabolism in the liver are key contributors to the development of liver fibrosis and injury [63–65]. Studies have shown that PPI and PPII can induce severe hepatotoxicity in a dose-dependent manner by disrupting lipid and energy metabolism pathways. These disruptions are mediated through mitochondrial dysfunction and the inhibition of key enzymes involved in lipid metabolism [66].

PPI impairs mitochondrial function by inhibiting β-oxidation, preventing the metabolism of free fatty acids. As a result, free fatty acids are converted into triglycerides, leading to lipid accumulation in the liver. The peroxisome proliferator-activated receptor (PPAR) signaling pathway, essential for lipid metabolism, is significantly affected by PPI. Both PPARα, which regulates genes involved in lipid clearance, and PPARγ, which promotes adipocyte differentiation and glucose uptake, are inhibited. This suppression reduces the activity of key enzymes in the PPAR pathway, including stearoyl-CoA desaturase-1, acyl-CoA oxidase 1, and CD36 (a fatty acid transporter), highlighting the pathway’s critical role in lipid metabolism and accumulation.

Furthermore, proteomic and transcriptomic analyses in human liver cell lines (L-02) and zebrafish models have revealed that exposure to PPI and PPII disrupts the cholesterol biosynthesis pathway, as evidenced by the downregulation of critical enzymes such as HMG-CoA reductase (HMGCR) and squalene epoxidase.

### 3.2 Hematotoxicity

Drug-induced hemolysis refers to the rupture and destruction of red blood cells (RBCs) caused by certain drugs or their metabolites, resulting in the release of hemoglobin into the plasma [67]. Common mechanisms include oxidative stress, immune reactions, direct toxicity, and interference with metabolic pathways [68,69]. Hemolysis can result in clinical symptoms such as anemia, jaundice, and kidney dysfunction, and in severe cases, it may be life-threatening [70–72].

#### 3.2.1 Clinical manifestations

Studies have shown that no hemolysis occurs when the concentration of total Rhizoma Paridis saponins is ≤0.01 g/L. However, when the concentration exceeds 0.01 g/L, hemolysis occurs, and its intensity is dose-dependent [73]. Fang and colleagues, using visual inspection and spectrophotometry, evaluated the hemolytic activity of total Rhizoma Paridis saponins, pennogenin saponins, and dioscin. They found that pennogenin saponins exhibited stronger hemolytic effects than dioscin, suggesting that pennogenin saponins are the primary hemolytic compounds in Rhizoma Paridis. Additionally, research indicates that compounds with similar structures, such as PPI, PPII, and PPD, also exhibit strong *in vitro* hemolytic activity.

**Figure 3:**
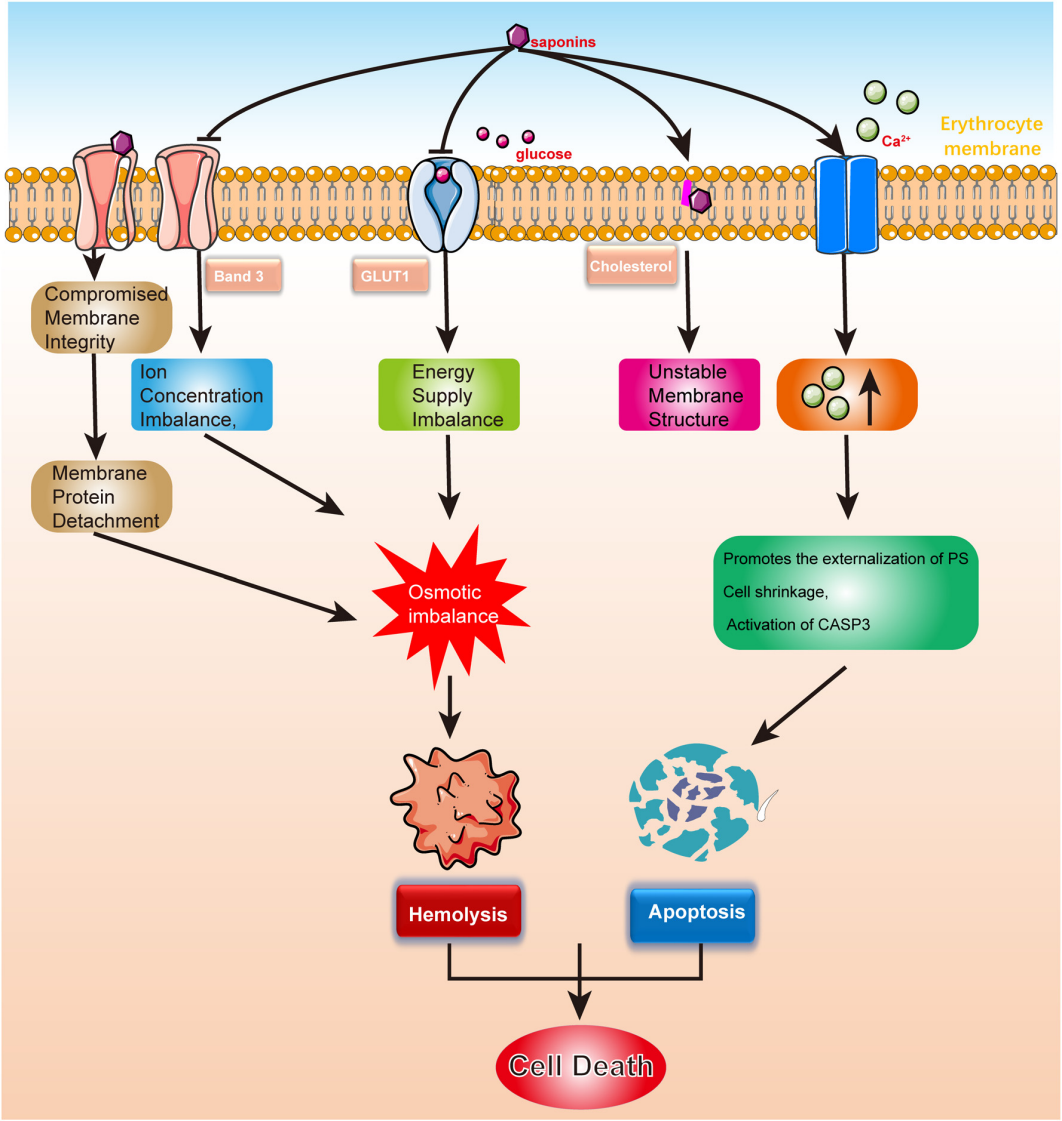
The mechanism of blood toxicity induced by Rhizoma Paridis saponins.

#### 3.2.2 Mechanisms of hematotoxicity (Figure 3)

##### 3.2.2.1 Binding to cholesterol on RBC membrane

The steroid saponins in Parnassia compounds may bind to cholesterol on the erythrocyte membrane, forming insoluble complexes. This binding enhances lipid peroxidation reactions within the membrane, leading to membrane structural instability. As a result, the normal osmotic pressure of the erythrocytes is disrupted, ultimately causing hemolysis due to the rupture of the RBCs [74].

#### 3.2.3 Binding to anion channels and inhibiting GLUT1

Anion channels, such as Band 3, are essential proteins on the RBC membrane that maintain the ionic balance between the intracellular and extracellular environments [75]. Studies have shown that saponins from Rhizoma Paridis, particularly PPII, can bind to Band 3 and inhibit its transport activity. This disruption alters the ion transport function of the RBC membrane, resulting in an imbalance of ion concentrations across the membrane. As a consequence, intracellular osmotic pressure increases, leading to RBC rupture and hemolysis. Additionally, PPII may alter the structure of Band 3 and other membrane proteins, thereby damaging the RBC cytoskeleton and causing the loss of normal cell morphology and function. This damage compromises the structural integrity of the RBC membrane, causing partial detachment of membrane proteins and increasing the cell’s susceptibility to hemolysis.

Furthermore, PPII competitively inhibits GLUT1, reducing glucose transmembrane transport. This inhibition impairs the energy supply to RBCs and disrupts intracellular osmotic pressure, leading to cell swelling and rupture [76]. Studies have demonstrated that GLUT1 inhibition promotes hemolysis, further supporting the mechanism by which PPII induces hemolysis through the suppression of glucose transport.

The interaction between anion channels and GLUT1 may jointly regulate RBC morphology and function by forming protein complexes, such as the Band 3–GLUT1 interaction. By binding to both channels, PPII may alter their structure or function, disrupting the transport of ions and glucose, which exacerbates the increase in intracellular osmotic pressure, promoting RBC swelling and rupture [77].

#### 3.2.4 Potential mechanism of polyphyllin D (PD)-induced hemolysis

Recent studies have demonstrated that PD exhibits significant blood toxicity, capable of inducing hemolysis and apoptosis in human RBCs. The mechanism underlying PD-induced hemolysis begins with the activation of calcium channels on the RBC membrane, leading to an increase in intracellular calcium ion concentration. This calcium influx promotes the externalization of phosphatidylserine, cell shrinkage, and the activation of caspase-3, which are hallmark features of apoptosis (eryptosis). In addition, PD directly affects the RBC membrane, increasing its permeability and facilitating the exchange of calcium ions between the intracellular and extracellular environments. This further amplifies the activation of the calcium-dependent apoptotic pathway. Due to its strong hemolytic and cytotoxic effects, PD’s clinical application is limited. Therefore, future research should focus on developing more efficient drug delivery systems to mitigate its toxicity to RBCs while preserving its anticancer potential [78].

### 3.3 Gastrointestinal toxicity

#### 3.3.1 Clinical manifestations

Gastrointestinal toxicity induced by Rhizoma Paridis is a significant concern in its clinical application. Excessive intake of Rhizoma Paridis can result in gastrointestinal side effects, including nausea, vomiting, and diarrhea. Studies have shown that a single administration of Rhizoma Paridis saponins inhibits gastric emptying in a dose-dependent manner without affecting intestinal transit in mice, indicating gastric irritation [79].

#### 3.3.2 Mechanisms of gastrointestinal toxicity

Research has demonstrated that the cytotoxicity of saponins is primarily due to their membrane toxicity, which may result from the depletion of cholesterol in cell membranes [74]. When saponins reach a certain concentration in the small intestine, they increase the permeability of intestinal mucosal cells, inhibit active nutrient transport, and facilitate the absorption of substances that are normally impermeable to the intestinal epithelium [80]. Despite these findings, the precise mechanisms underlying the gastrointestinal irritation caused by Rhizoma Paridis remain poorly understood. Further research is needed to ensure its effective and safe clinical use.

### 3.4 Cardiovascular toxicity

#### 3.4.1 Clinical manifestations

Drug-induced cardiovascular side effects contribute significantly to morbidity and mortality rates [81]. Clinical reports indicate that excessive intake of Rhizoma Paridis can lead to cardiotoxic effects, manifesting as symptoms such as arrhythmias and muffled heart sounds. Furthermore, studies have demonstrated that three saponins – PPⅠ, PPⅡ, and PPⅦ – strongly inhibit the proliferation of cardiovascular cells, including H9c2, HUVEC, and HCMEC. Notably, PPⅠ has shown significant cardiotoxicity in both zebrafish and mouse models, suggesting a potential risk of cardiovascular damage [82].

#### 3.4.2 Mechanisms of cardiovascular toxicity

The cardiovascular toxicity of Rhizoma Paridis is primarily associated with a significant inhibition of cell viability, migration, invasion, and angiogenesis. The underlying mechanisms of PPI-induced cardiovascular toxicity occur via two major pathways: inhibition of angiogenesis and induction of endothelial cell apoptosis.

First, PPI suppresses the tyrosine phosphorylation of vascular endothelial growth factor receptor 2 (VEGFR2), thereby reducing VEGF signaling. This, in turn, inhibits endothelial cell proliferation and migration through several downstream signaling pathways, including PI3K/Akt/mTOR, Src/eNOS, p38, and PLCγ/ERK/MEK. These effects ultimately block new blood vessel formation. Additionally, PPI inhibits the JAK2/STAT3 signaling pathway, further suppressing endothelial cell activity and reducing angiogenesis.

Second, PPI induces apoptosis in cardiovascular cells, as evidenced by the release of lactate dehydrogenase, morphological changes such as apoptotic body formation and chromatin condensation, and cell cycle arrest, particularly in the S and G2/M phases, leading to cell death. At the molecular level, PPI upregulates Bax, downregulates Bcl-2, and activates caspase-9, thereby initiating the mitochondrial pathway to induce apoptosis.

**Figure 4:**
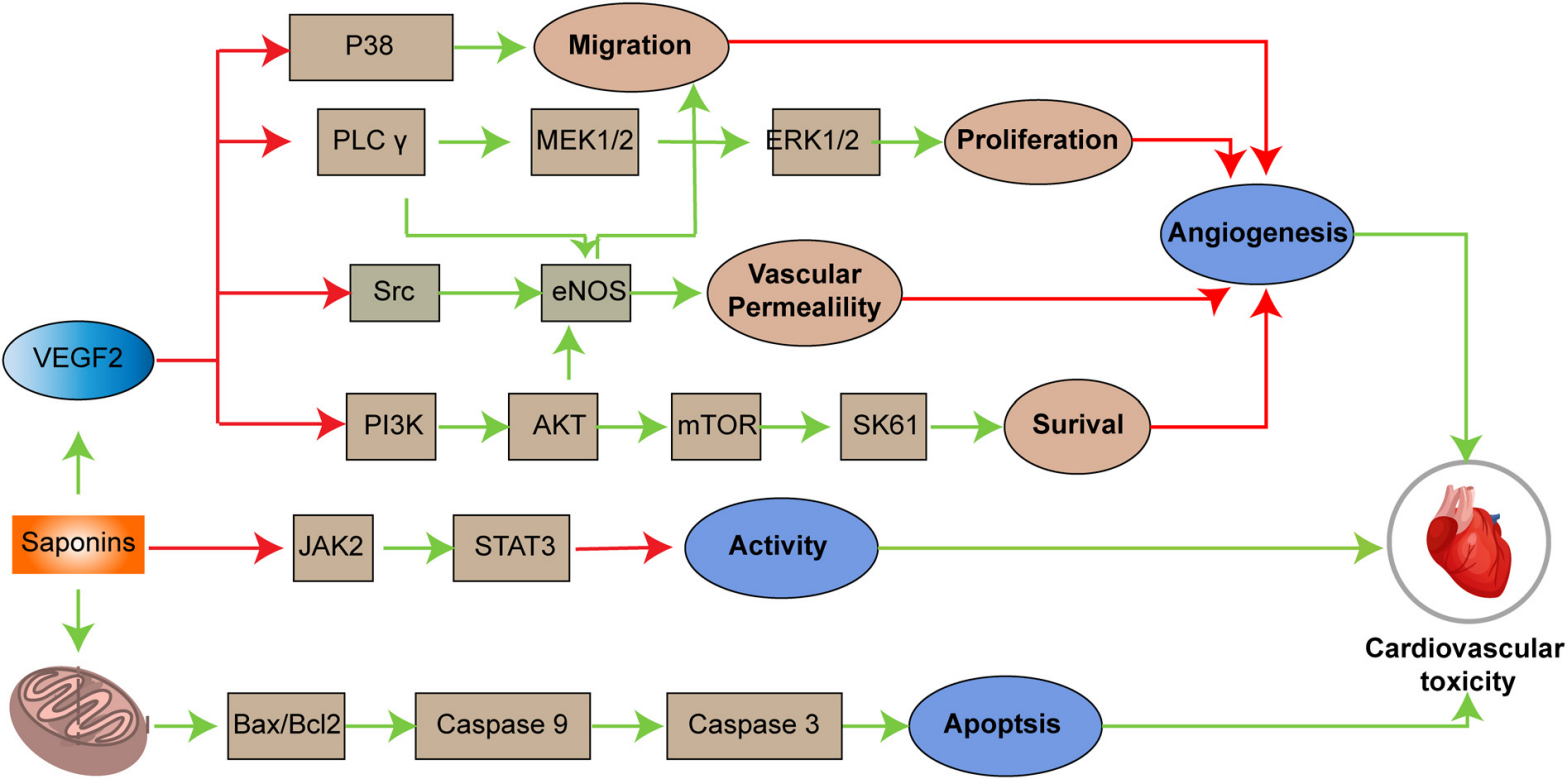
The mechanism of cardiovascular toxicity induced by Rhizoma Paridis saponins.

In summary, PPI likely exerts cardiovascular toxicity through the combined effects of multiple signaling pathways, inhibiting angiogenesis and inducing endothelial cell apoptosis (Figure 4) [83].

### 3.5 Reproductive toxicity

Shen et al. investigated the effects of Rhizoma Paridis on sperm viability through *in vitro* sperm incubation. Their study found that pennogenin saponins and dioscin, isolated and purified from Rhizoma Paridis, significantly reduced sperm viability in mice. At a final concentration of 40 mg/L, dioscin completely killed all sperm, while some sperm remained viable when exposed to pennogenin saponins at the same concentration [84]. However, it is important to note that Rhizoma Paridis has not undergone systematic reproductive toxicity studies in accordance with international and national guidelines [85]. Most existing studies have focused on reproductive organ toxicity, indicating potential reproductive risks but failing to precisely characterize the types and severity of toxicity (e.g., fertility toxicity, early embryonic development toxicity, fetal development toxicity, perinatal toxicity, and maternal toxicity). As a result, current research offers limited guidance for the clinical application of Rhizoma Paridis, highlighting the need for further studies.

### 3.6 Long-term toxicity

Long-term toxicity studies of Rhizoma Paridis saponins have shown significant chronic toxicity, particularly affecting the liver and gastrointestinal systems. High doses (350 mg/kg) of Rhizoma Paridis saponins in rats led to weight loss, reduced food and water intake, indicating impaired gastrointestinal function. Prolonged exposure caused liver cell damage, oxidative stress, and elevated liver markers (ALT and AST), with the high-dose group showing the most severe effects. The medium and low doses (50 and 100 mg/kg) also caused some liver damage [86].

Although research on pulmonary toxicity is limited, other saponins from TCM suggest lung damage mechanisms involving inflammation and oxidative stress. Further studies are needed to assess pulmonary risks of Rhizoma Paridis saponins.

However, liver function in all dose groups returned to normal after a 4-week withdrawal, suggesting the chronic toxicity of Rhizoma Paridis saponins may be reversible, likely due to compensatory mechanisms like antioxidant system activation and tissue repair.

### 3.7 Summary

In summary, the hepatotoxicity, hemolysis, gastrointestinal toxicity, and cardiovascular toxicity associated with Rhizoma Paridis are primarily attributed to its saponin content. However, the severity and specific nature of these toxic effects vary among the different saponins present in Rhizoma Paridis, including Polyphyllin I, Polyphyllin II, and Polyphyllin Ⅶ [82,87–89] (Table 2).

**Table 2:** Comparison of toxicity profiles of different Rhizoma Paridis saponins

Characteristic	Polyphyllin I	Polyphyllin II	Polyphyllin VII
Toxicity intensity	Strongest	Moderate	Weakest
Toxicity targets	Liver, cardiovascular system, RBCs, kidneys, lungs, sperm	Liver, kidneys, heart, sperm	Liver, kidneys, sperm
Organ toxicity features	Liver: severe liver toxicity, vacuolization, necrosis; heart: Severe cardiovascular toxicity, impacts heart rate, promotes cardiac cell apoptosis; Kidneys: Mild toxicity, no renal edema; lungs: Thickened alveolar walls	Liver: Hepatocyte necrosis; kidneys: Mild tubular damage	Liver: Mild hepatocyte vacuolization; kidneys: Slight lipid metabolism disorder
Experimental models	Cells (heart, liver, kidney)	Cells (heart, liver, kidney)	Cells (heart, liver, kidney)
	Zebrafish	Zebrafish	Zebrafish
	LC_50_ 121 ng/mL	LC_50_ 213 ng/mL	LC_50_ 570 ng/mL
	LC_10_ 109 ng/mL	LC_10_ 178 ng/mL	LC_10_ 456 ng/mL
	MNLC 99 ng/mL	MNLC 146 ng/mL	MNLC 357 ng/mL
	Mice LC_50_ 24.5 mg/kg	No data available	No data available
Conclusion	Significant toxicity with multiple targets, requires strict dose control, suitable for short-term applications, clinical use must be cautious, especially at high doses	Relatively mild toxicity with concentrated targets, suitable for combination therapy or single-drug development	Lowest toxicity, high potential for application, suitable for further clinical studies
References	[87–90]	[87–89]	[87,89]

The inclusion of a comparison of the toxicity of different saponins is crucial because it helps to better understand the varying risks associated with Rhizoma Paridis. These saponins have unique pharmacological properties that contribute to different toxicity mechanisms. By identifying the specific saponins present in a formulation, clinicians can make more informed decisions regarding dosage, treatment regimens, and monitoring strategies. This comparison also highlights the need for individualized treatment plans, tailored to the specific toxicity risks posed by the particular saponin content, thereby optimizing therapeutic outcomes while minimizing adverse effects.

## 4 Toxicity prevention and treatment of Rhizoma Paridis

Understanding the toxicity of medicinal herbs and developing effective prevention and detoxification strategies are crucial in the application of TCM. Effective toxicity management not only enhances therapeutic efficacy but also ensures the safety of the treatment. This section reviews both traditional and modern detoxification methods for Rhizoma Paridis, critically evaluating their scientific potential and limitations, with the aim of providing guidance for its safe clinical use.

### 4.1 Considerations in the use of Rhizoma Paridis

Existing evidence suggests that Rhizoma Paridis possesses a certain degree of toxicity, necessitating careful attention to dosage control and appropriate usage methods.

#### 4.1.1 Dosage control

Effective management of Rhizoma Paridis dosage is critical to mitigate potential toxicity risks. Clinical studies underscore the importance of strict dosage control, typically recommending a range of 3–9 g per administration. However, individualization of dosage is paramount, considering factors such as the patient’s age, body weight, health status, and tolerance levels. Overdosing on Rhizoma Paridis can lead to severe adverse reactions, particularly hepatotoxicity characterized by liver damage. Monitoring of liver enzymes and other relevant biomarkers during treatment is advisable to promptly detect and manage any emerging toxicity.

#### 4.1.2 Contraindicated populations

Rhizoma Paridis should be avoided by pregnant and breastfeeding women, as well as individuals with impaired liver or kidney function. Populations with compromised metabolic and detoxification abilities, such as the malnourished or those with chronic diseases, are at heightened risk of toxic reactions and should use this herb with caution. Additionally, individuals with a history of allergies should avoid its use.

### 4.2 Methods of reducing toxicity

TCM has developed extensive strategies for managing toxicity, primarily through herbal combinations, preparation techniques, and formulation adjustments that reduce toxicity while enhancing efficacy.

#### 4.2.1 Herb pairing for toxicity reduction

In TCM, herb pairing is a crucial strategy for enhancing therapeutic effects and mitigating toxicity [90–92]. Rhizoma Paridis is often combined with other herbs to reduce its toxic effects. For example, pairing it with *Glycyrrhiza* and *Curcuma longa* can alleviate liver toxicity. Modern pharmacological studies suggest that the flavonoids in *Glycyrrhiza* can repair liver tissue by reducing inflammation, enhancing antioxidant enzyme activity, and decreasing oxidative stress in the liver. Furthermore, *Glycyrrhiza* saponins may also reduce oxidative stress and inflammation by inhibiting ROS production and regulating the NF-κB pathway, potentially reducing liver toxicity [93]. However, the specific detoxification mechanisms of *Glycyrrhiza* in combination with Rhizoma Paridis require further investigation.

*C. longa* has also shown potential in mitigating the toxicity of Rhizoma Paridis. Research indicates that combining *C. longa* with Rhizoma Paridis may alleviate toxicity in both the liver and lungs. The detoxification mechanism of *C. longa* likely involves the reduction of ROS, suppression of inflammatory markers, and activation of the Nrf2/HO-1 detoxification pathway. However, this mechanism needs further clinical and preclinical validation [94].

#### 4.2.2 Processing methods for toxicity reduction

The processing of medicinal herbs plays a crucial role in reducing toxicity and enhancing efficacy. Proper preparation methods can decrease the toxicity of Rhizoma Paridis without compromising its therapeutic effects, while improper processing may exacerbate toxicity. For instance, vinegar processing of Rhizoma Paridis has been shown to reduce its gastrointestinal irritant effects. While traditional processing methods have demonstrated some effectiveness in reducing toxicity, they are not standardized, and scientific validation of their detoxifying effects is limited.

#### 4.2.3 Application of modern scientific technologies

Modern pharmacology has provided important insights into the toxicological mechanisms of Rhizoma Paridis, facilitating the development of more precise preventive measures. Advanced toxicological evaluation techniques allow for a comprehensive understanding of Rhizoma Paridis’s toxic properties before clinical use, helping to minimize adverse reactions. The use of animal and cell models has expanded the scope of toxicity assessment, offering critical guidance for safe usage.

Innovative pharmaceutical technologies, such as microemulsions and liposomes, significantly improve the bioavailability of drugs while reducing liver damage. For instance, studies have demonstrated that encapsulating Saikosaponin D in liposomes can reduce its toxicity [95]. This approach may also provide a potential strategy for mitigating the toxicity of Rhizoma Paridis.

Additionally, modern research suggests that antioxidant supplements, such as Vitamin C and Vitamin E, can enhance liver detoxification and reduce oxidative stress associated with Rhizoma Paridis toxicity [96,97]. While these adjunctive treatments show promise in mitigating toxicity, further clinical data are needed to determine the best combinations of auxiliary herbs for use with Rhizoma Paridis.

### 4.3 Detoxification methods

When toxicity occurs, timely and effective detoxification measures are crucial to minimize damage. Upon poisoning, the use of Rhizoma Paridis must be immediately discontinued. Common detoxification methods, both traditional and modern, help reduce toxin absorption and promote elimination, thereby alleviating poisoning symptoms.

#### 4.3.1 Traditional detoxification methods

TCM often employs herbal remedies for detoxification, including *Scutellaria baicalensis*, immature bitter orange, etc. [98,99]. *S. baicalensis* is known for its antioxidant, anti-inflammatory, and cell-regulating properties, which can alleviate liver damage caused by toxins. Modern pharmacological studies indicate that *Scutellaria* and its active compounds (such as baicalin) can synergize with other drugs to enhance efficacy, reduce toxicity, or overcome drug resistance [100,101]. Moreover, Chai Hu saponins can prevent liver toxicity induced by acetaminophen through inhibition of NF-κB and STAT3 signaling pathways. Licorice Decoction combined with rice vinegar and ginger juice are used for detoxifying Rhizoma Paridis poisoning [102].

Decoction therapy is a traditional detoxification method in Chinese medicine, with a rich history of clinical application. For example, in cases of poisoning from certain cardiac drugs (e.g., digoxin), the use of Shengmai San can help protect heart function [103]. Classic formulas such as Jiedu Tang, Huang Lian Jiedu Tang, and Da Cheng Qi Tang are commonly used to alleviate liver damage [104]. However, there is currently no specific formula available for liver injury caused by Rhizoma Paridis, indicating the need for further research to develop effective detoxification remedies. Decoctions, by gently promoting detoxification and facilitating the metabolism and excretion of toxins, are widely used in traditional clinical practice. However, their efficacy is often influenced by factors such as the patient’s constitution and the ratio of medicinal ingredients. Furthermore, a lack of scientific evidence for precision treatment calls for continued investigation to verify their effectiveness and clinical feasibility.

#### 4.3.2 Modern detoxification methods

Advancements in modern medicine have introduced chemical detoxifiers and pharmaceutical technologies that offer new avenues for detoxification. These methods are typically more controllable and scientifically validated.

Common general detoxification treatments include gastric lavage, purgation, and the administration of diluted acetic acid. Gastric lavage effectively reduces toxin concentration in acute poisoning cases. For example, in cases of colchicine poisoning, where there is no clear distinction between toxic and lethal doses, clinicians often consider gastrointestinal decontamination or gastric lavage using activated charcoal [105].

Chemical detoxifiers, such as *N*-acetylcysteine (NAC), replenish glutathione levels and restore the liver’s antioxidant capacity, making it a cornerstone treatment for liver toxicity. Cholesterol inhibitors, such as lovastatin, have been shown to mitigate saponin-induced membrane damage by inhibiting HMGCR, thereby regulating cholesterol metabolism and stabilizing cholesterol levels within cell membranes. This stabilization helps alleviate hemolysis caused by saponins. Additionally, lovastatin provides hepatoprotective effects by reducing oxidative stress and inflammatory responses, thereby attenuating saponin-induced hepatotoxicity [106].

### 4.4 Comparison and integration of traditional and modern methods

Traditional detoxification methods, such as herb pairing and processing, offer advantages in the management of chronic toxicity, including lower costs and a wealth of historical experience. However, these methods lack molecular mechanism validation and their efficacy can vary across individuals. In contrast, modern methods like NAC and magnesium isoglycyrrhizinate offer more precise control over ROS and metabolic pathways, providing more effective solutions, particularly in the management of acute toxicity. However, modern methods are often costly and technically complex, limiting their widespread application in resource-constrained regions [107–116] (Table 3). Future research should explore the integration of traditional and modern approaches, such as combining herbal pairings (e.g., *Glycyrrhiza*, *C. longa*) with modern pharmaceutical technologies (e.g., nanomedicines), to achieve synergistic detoxification and promote the combined use of both strategies.

**Table 3:** A comparative analysis of traditional and modern detoxification methods

Aspect	Traditional methods	Modern methods
Mechanism	Based on herb combinations and processing	Using molecular biology, enzyme inhibition, and modern detox mechanisms
Efficacy	Relies on experience and intuition, adjusts overall effects	Quality and toxicity controlled via instrument testing and standardization
Processing time and efficiency	Long process, experience-based	Fast, standardized, easy to monitor quality
Advantages	Holistic approach, personalized treatment, accumulated historical experience	Scientifically-based, quantifiable detox effects, fast, stable efficacy
Disadvantages	Lack of scientific validation, unstable efficacy, long treatment time	High cost, potential introduction of new toxicity
References	[107–112]	[108,113–116]

## 5 Evaluation of TCM toxicity

Although current toxicological studies provide a theoretical foundation for the clinical application of Rhizoma Paridis, they remain incomplete. Therefore, further research on its toxicity is essential. This section summarizes key aspects of toxicity studies for TCM, with the aim of offering more comprehensive guidance for the safe and effective use of Rhizoma Paridis.

### 5.1 Key aspects of toxicity studies

Key aspects of toxicity studies are essential for assessing the safety of a drug throughout its development, from preclinical to clinical stages. One critical area is acute and chronic toxicity, which involves evaluating the toxic effects of Rhizoma Paridis at varying doses and durations to identify both immediate and long-term risks. Another key aspect is organ-specific toxicity, where researchers examine the effects of Rhizoma Paridis on vital organs such as the liver, kidneys, and heart to assess potential organ damage. Reproductive toxicity assessments are also crucial, as they evaluate the herb’s impact on fertility and fetal development to ensure it does not adversely affect the reproductive system. Additionally, understanding the cytotoxic mechanisms at the cellular level is important for exploring how Rhizoma Paridis induces toxicity within cells, providing insights into how its harmful effects might be mitigated. Finally, studying drug interactions is vital for analyzing how Rhizoma Paridis interacts with other medications, as these interactions can influence its efficacy and safety profile, potentially leading to unexpected adverse effects. Together, these comprehensive toxicity studies form a solid foundation for the safe and effective clinical use of Rhizoma Paridis.

### 5.2 Research methods and techniques

The complexity of TCM components and their diverse biological targets necessitates the development of specialized safety assessment techniques tailored to the unique characteristics of TCM. Establishing these techniques is essential for conducting large-scale toxicity studies and achieving a comprehensive understanding of the potential risks associated with TCM.

#### 5.2.1 Animal experiments

In TCM toxicity evaluations, rats and mice are commonly used for acute and subacute toxicity tests [117–119]. However, due to the complexity and multiple targets of TCM components, traditional mammalian models present challenges such as high costs, long testing durations, and limitations in high-throughput screening capacity.

As an alternative, the chick embryo model has gained popularity due to its ease of manipulation, relative transparency, and accessibility, making it an ideal platform for high-throughput screening. The physiological and pathological changes in chick embryos can be visualized using techniques such as candling, staining, and imaging, bridging the gap between *in vivo* and *in vitro* studies. This has established chick embryos as valuable tools for pharmacodynamics and toxicity research in TCM [120].

In recent years, zebrafish have become widely used in TCM toxicity studies. The advantages of the zebrafish model include its low cost, short lifecycle, ease of high-throughput screening, and exemption from strict ethical regulations before 5 days post-fertilization [121]. Studies have demonstrated the applicability of zebrafish in assessing various toxicities, including acute, hepatic, cardiac, renal, developmental, neurological, gastrointestinal, immunological, and ototoxicity [122]. For instance, zebrafish models have been used to evaluate the nephrotoxicity of aristolochic acid, confirming the model’s stability and accuracy in TCM toxicity screening [123]. Zebrafish have also been employed to assess the toxicity of artemisinin compounds [124]. However, it is important to note that physiological differences limit the zebrafish model’s comprehensive application [125]. Researchers have applied zebrafish models to study the toxicity of Rhizoma Paridis, providing evidence of its toxic properties.

Overall, integrating traditional mammalian models, chick embryos, and zebrafish provides a multi-tiered evaluation system for Rhizoma Paridis toxicity research. The combined use of these models offers a more comprehensive understanding of the toxicological properties of Rhizoma Paridis, thereby supporting its safe and effective clinical application.

#### 5.2.2 Cell experiments

Developing cell models that replicate *in vivo* responses is essential for advancing scientific research and improving ethical standards [126]. These *in vitro* models allow researchers to conduct drug screening and toxicity assessments more efficiently, reducing the ethical concerns and costs associated with animal experiments. For example, cardiomyocytes derived from embryonic stem cells or human-induced pluripotent stem cells are increasingly used to predict cardiotoxicity in the early stages of drug development [127].

Furthermore, the advancement of organoid technology provides new opportunities for evaluating the toxicity of TCM. Organoids are three-dimensional miniature organs formed through the self-organization of stem cells and can closely mimic the structure and function of actual organs. Researchers have successfully created cardiac tissue models to assess the impact of TCM on heart health, and similar platforms have been developed to evaluate TCM-induced nephrotoxicity using organoid technology and advanced cell models [128,129]. These platforms offer efficient and precise tools for studying the toxicity of Rhizoma Paridis.

By integrating traditional mammalian models with advanced *in vitro* systems, a comprehensive evaluation framework can be established for the toxicity research of Rhizoma Paridis. The application of these models will provide deeper insights into the toxicological properties of Rhizoma Paridis, supporting its safe and effective clinical use.

## 6 Discussion

Increasing evidence supports the potential of natural medicines in disease treatment [130–138]. For example, Tu Youyou successfully extracted artemisinin from Chinese herbal medicine and applied it to treat malaria, saving millions of lives [139].

Rhizoma Paridis, a TCM with significant clinical potential, has shown promising preclinical therapeutic effects. Extensive research has demonstrated its pharmacological activities, including antitumor, anti-inflammatory, and antifibrotic effects [140–142]. For instance, preclinical studies have shown that PPII not only inhibits tumor growth but also enhances sensitivity to treatment by inducing pyroptosis in tumor cells, exhibiting strong anticancer effects in models such as lung cancer [143–145].

However, despite its great therapeutic potential, the toxicity of Rhizoma Paridis remains a major obstacle to its widespread application, particularly in relation to liver, cardiovascular, and gastrointestinal side effects [146]. Existing research mainly focuses on acute toxicity, while data on chronic toxicity are insufficient. Long-term use, particularly at high doses or over extended periods, may lead to additional adverse effects. Therefore, further investigation into its chronic toxicity is necessary to clarify the potential long-term risks to human health. Despite these concerns, when used within the recommended dosage range, Rhizoma Paridis demonstrates significant therapeutic effects, especially in treating cancer and immune system disorders. In fact, Rhizoma Paridis and its formulations have been used clinically with favorable outcomes. For example, Loulian capsules for primary liver cancer and Jinfukang oral liquid for primary non-small cell lung cancer have shown positive results. Clinical studies indicate that when administered at appropriate doses, the toxicity of Rhizoma Paridis is minimal, and it can effectively alleviate related symptoms. Therefore, it is crucial for clinicians to precisely control the dosage, develop individualized treatment plans, and closely monitor patients’ physiological responses to ensure safety. However, we also highlighted the need for additional clinical trials and randomized controlled studies to further confirm the efficacy and safety of Rhizoma Paridis, ensuring its rational use in clinical settings. We believe that such studies are essential to establish clearer guidelines for its application in clinical practice.

As research into the toxicological mechanisms of TCM advances, the safety and efficacy of Rhizoma Paridis can be further improved, contributing to the development of TCM and standardizing its clinical applications. Current TCM toxicity research mainly relies on cell and animal experiments; however, with the development of modern technologies such as network pharmacology and machine learning, valuable insights into the toxicological mechanisms of TCM will emerge in the future [147].

Additionally, with the rapid development of modern drug delivery systems, innovative technologies are emerging that not only enhance the bioavailability of drugs but also reduce their toxicity. Poly lactic-co-glycolic acid (PLGA) nanoparticles, a drug delivery system known for its excellent biocompatibility and controlled release properties, can significantly improve the solubility and targeting of drugs. For Rhizoma Paridis, combining modern drug delivery technology, especially the co-loading of PPII and IR780 into PLGA nanoparticles, effectively overcomes the limitations of traditional drug delivery, such as solubility and targeting issues. This delivery system enables high concentrations of PPII and IR780 to accumulate at tumor sites, and, through the photothermal effect of IR780, precisely kill tumor cells under laser irradiation. Furthermore, the pyroptotic effect induced by PPII, in combination with the photothermal therapy from IR780, generates a synergistic effect, enhancing anticancer efficacy while reducing toxicity to normal cells, thereby improving the safety and effectiveness of the treatment [148].

By integrating modern drug delivery technology with the traditional therapeutic effects of TCM, we can maximize the therapeutic potential of Rhizoma Paridis while minimizing its toxicity, thus promoting its safe clinical application.

## 7 Future and prospectives

In summary, Rhizoma Paridis shows great potential as a medicinal herb, but more research is needed to fully understand its effects and safety. Future studies should focus on understanding how it works in the body and its long-term effects, especially since most research has looked at short-term toxicity. It is also important to study its chronic toxicity to ensure that it is safe for long-term use. Combining traditional knowledge with modern research methods like systems biology could help identify key pathways and biomarkers for both its benefits and risks. Additionally, strategies to reduce toxicity, such as improving how the herb is processed or combining it with other herbs, should be explored. Researchers should also look into how Rhizoma Paridis interacts with other drugs and its effects on drug metabolism. Ultimately, clinical trials will be necessary to confirm its safety and effectiveness in real-world use. By addressing these areas, future research can help ensure that Rhizoma Paridis is used safely and effectively in medical practice.
